# Supplementary figures and images for: The velvet family proteins mediate low resistance to isoprothiolane in Magnaporthe oryzae
Source: PLoS Pathog. 2023 Jun 5;19(6):e1011011. doi: 10.1371/journal.ppat.1011011 (PMC10270592; doi:10.1371/journal.ppat.1011011)

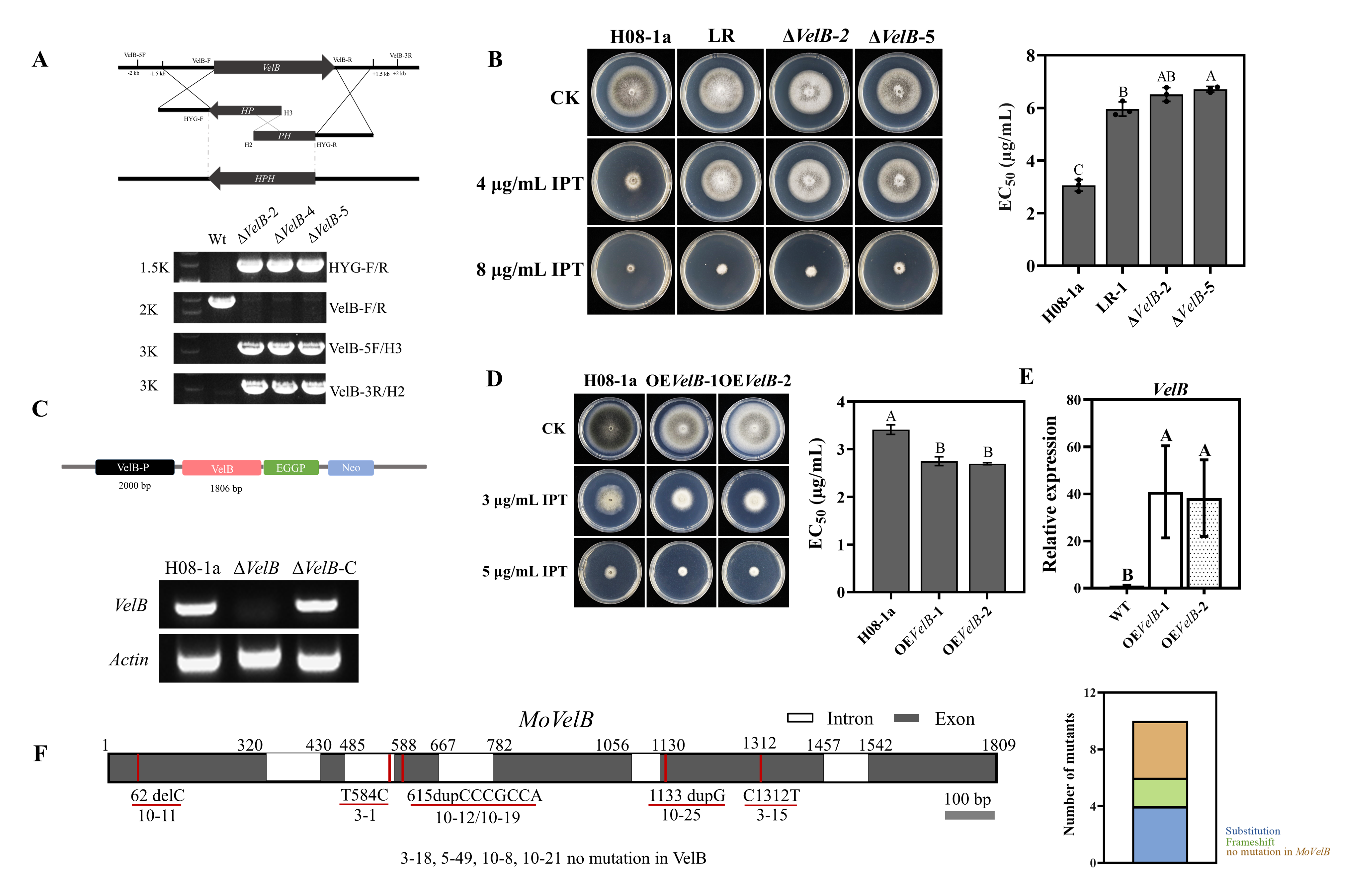

Supplement: S1 Fig — (A) Gene replacement strategy of MoVelB and identification of ΔVelB transformants by PCR. (B) Sensitivity analysis of ΔVelB transformants to IPT. Data presented are the mean ± SD (n = 3). Bars followed by the same letter are not significantly different according to a LSD test at P = 0.01. (C) Gene complementation strategy of MoVelB and identification of MoVelB complementation transformants by RT-PCR. (D) Sensitivity analysis of OEVelB transformants to IPT. Data presented are the mean ± SD (n = 3). Bars followed by the same letter are not significantly different according to a LSD test at P = 0.01. (E) Detection of MoVelB expression in OEVelB transformants by RT-qPCR. The MoActin gene was used as the internal reference for normalization. (F) Detection of variations of MoVelB in low resistant mutants. (TIF) [file ppat.1011011.s006.tif]

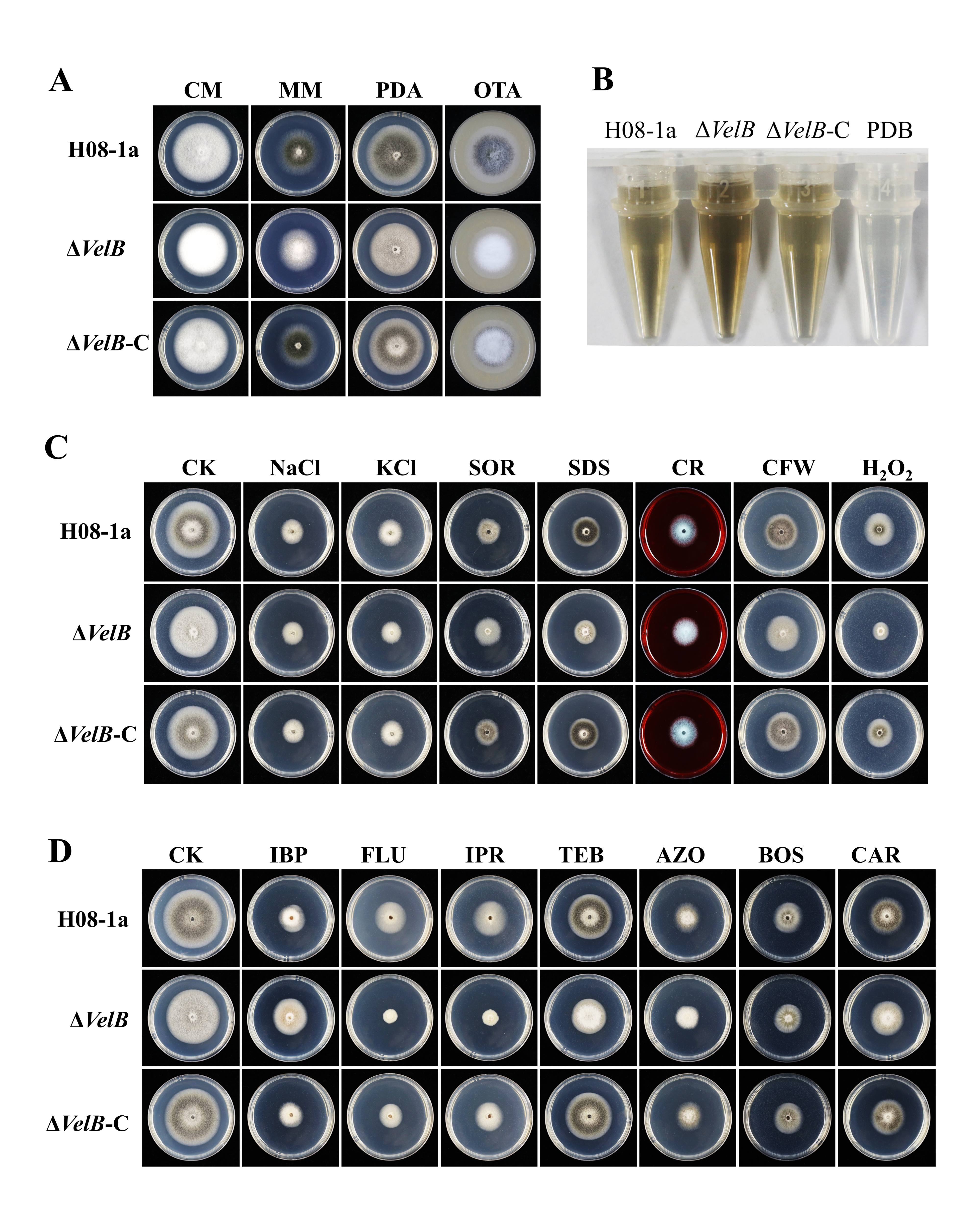

Supplement: S2 Fig — (A) Colony morphology of MoVelB knockout and complemented tansformants. (B) Melanin production in MoVelB knockout and complemented transformants. (C) Tolerance of MoVelB knockout and complemented transformants to different stresses. (D) Sensitivity of MoVelB knockout and complemented transformants to different fungicides. Strains were inoculated on different media with different stresses or fungicides at 27°C for 5 days. IBP, FLU, IPR, TEB, AZO, BOS, and CAR indicate the fungicides iprobenfos, fludioxonil, iprodione, tebuconazole, azoxystrobin, boscalid, and carbendazim, respectively. (TIF) [file ppat.1011011.s007.tif]

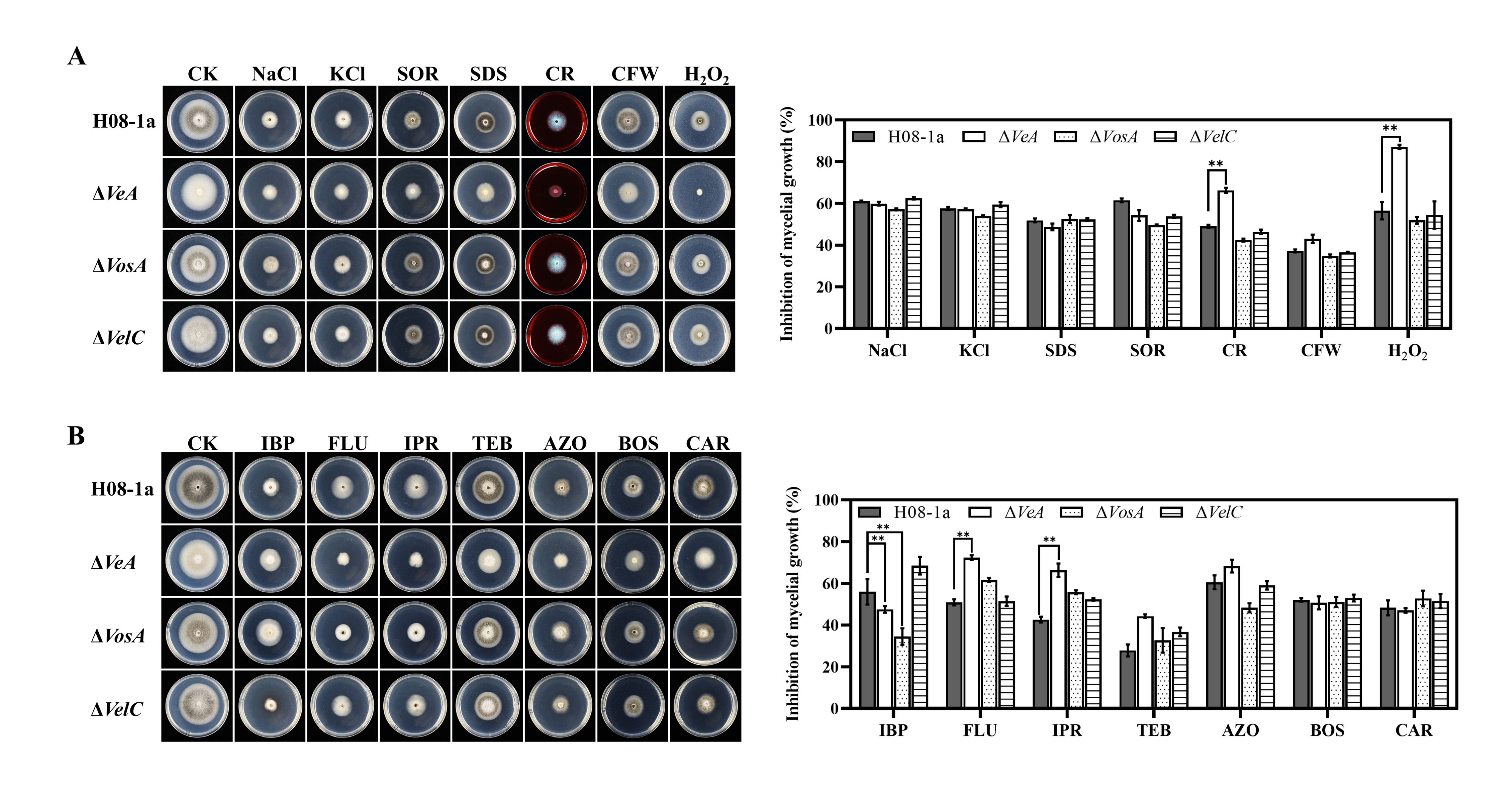

Supplement: S3 Fig — (A) The H08-1a, ΔVeA, ΔVosA and ΔVelC strains were incubated on PDA amended with different stress agents at 27°C for 5 days and statistical analysis of the growth inhibition rate. (B) The H08-1a, ΔVeA, ΔVosA and ΔVelC strains were incubated on PDA amended with different fungicides at 27°C for 5 days and statistical analysis of the growth inhibition rate. Data presented are the mean ± SD (n = 3). (TIF) [file ppat.1011011.s008.tif]

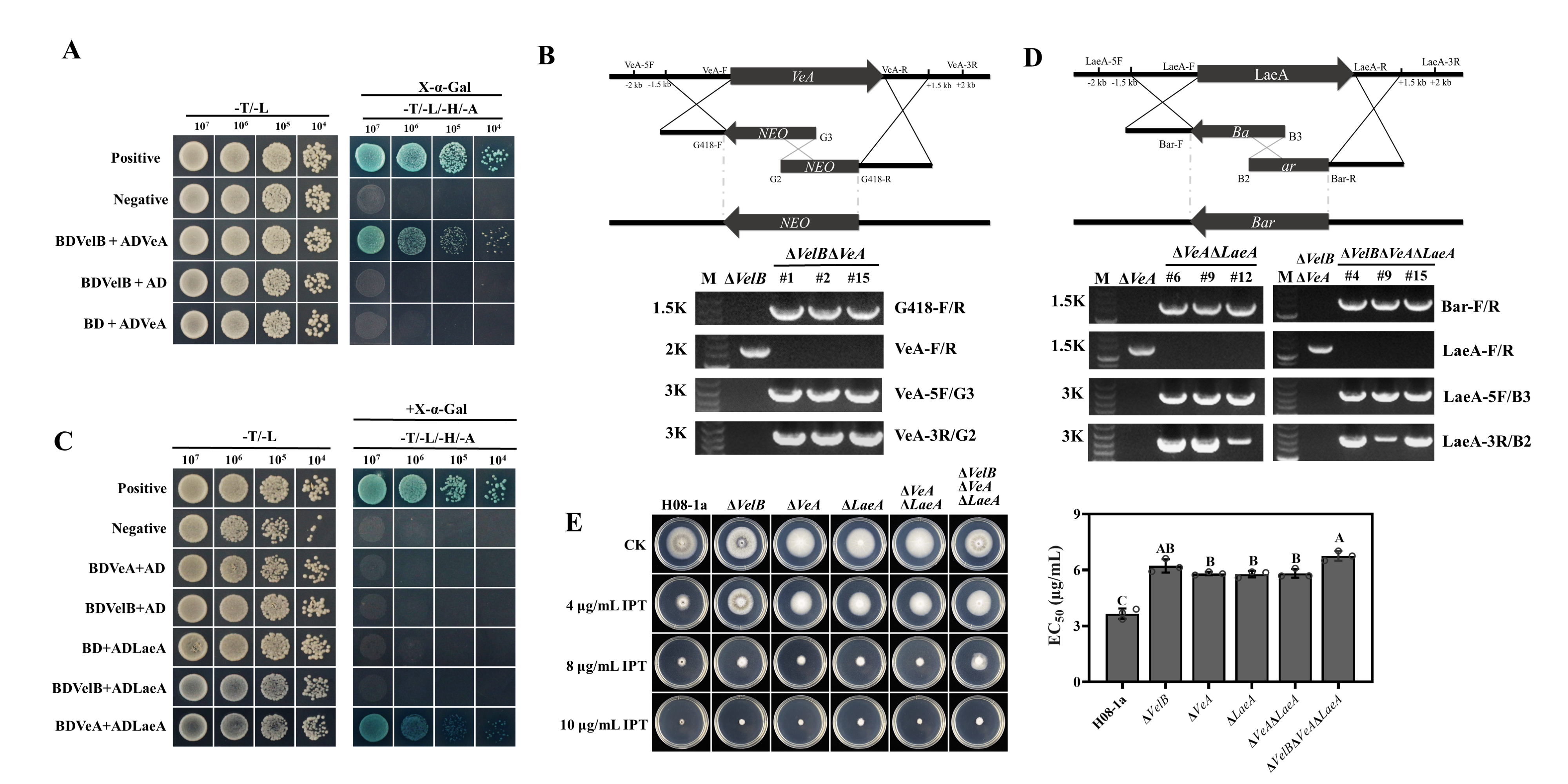

Supplement: S4 Fig — (A) The yeast two-hybrid (Y2H) assay revealed that MoVeA interacted directly with MoVelB. The plasmid pairs of pGADT7/pGBKT7-53 and pGADT7/pGBKT7-Lam served as the positive and negative controls, respectively. (B) Gene knockout strategy of MoVeA and identification of MoVelB and MoVeA double knockout transformants by PCR. (C) The yeast two-hybrid (Y2H) assay revealed that MoLaeA interacted directly with MoVeA, but not with MoVelB. (D) Gene knockout strategy of MoLaeA and identification of MoVeA and MoLaeA double knockout transformants, MoVelB, MoVeA and MoLaeA triple knockout transformants by PCR. (E) Sensitivity analysis of different velvet gene knockout transformants to IPT. Data presented are the mean ± SD (n = 3). Bars followed by the same letter are not significantly different according to a LSD test at P = 0.01. (TIF) [file ppat.1011011.s009.tif]

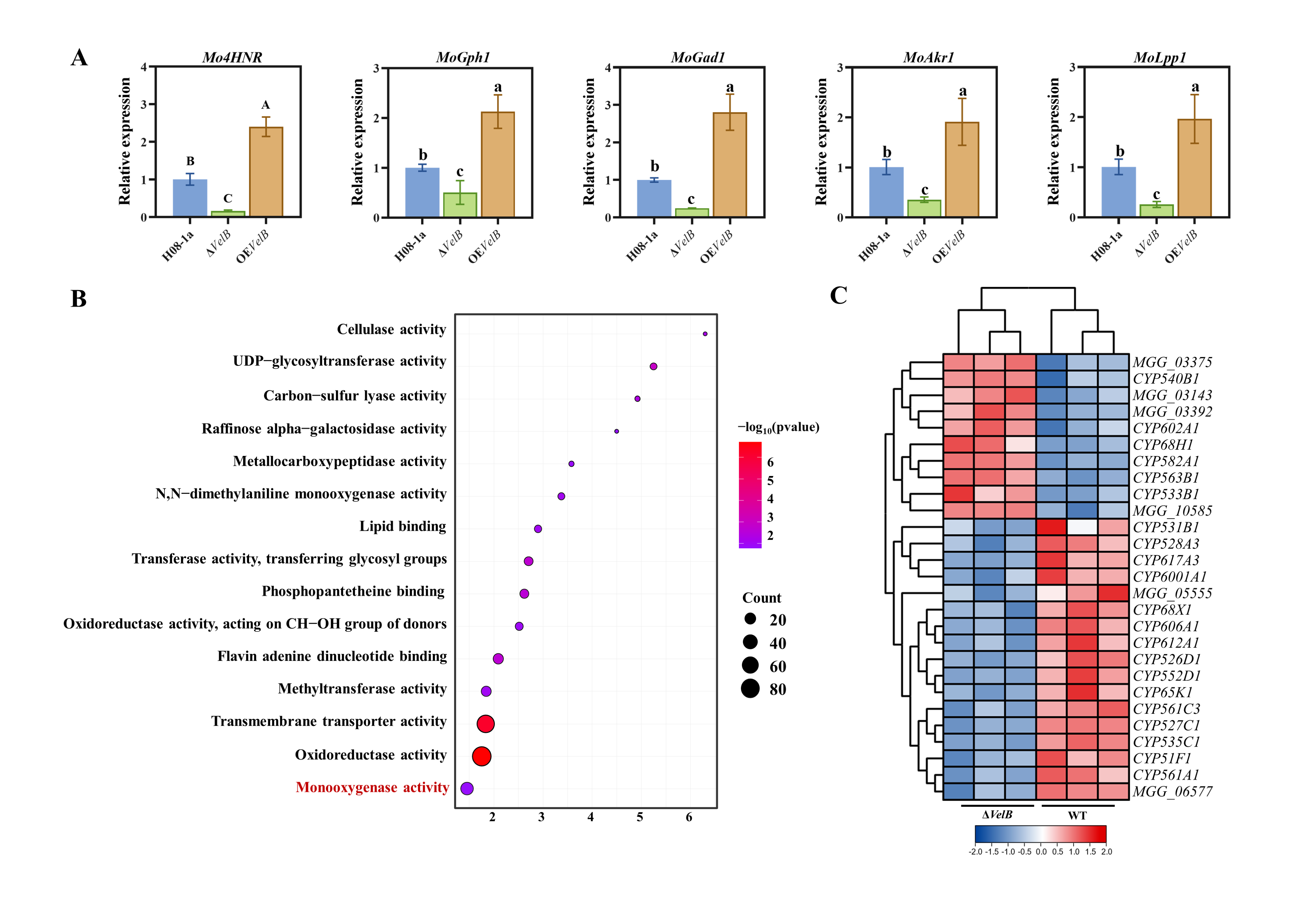

Supplement: S5 Fig — (A) Expression levels of five secondary metabolism-related genes in ΔVelB and OEVelB strains by RT-qPCR. The MoActin gene was used as the internal reference for normalization. (B) GO analysis of DEGs in ΔVelB compared to H08-1a. (C) Expression heat map of differentially expressed monooxygenases encoding genes in ΔVelB compared to H08-1a. (TIF) [file ppat.1011011.s010.tif]
