# Supplementary material for: The velvet family proteins mediate low resistance to isoprothiolane in Magnaporthe oryzae
Source: PLoS Pathog. 2023 Jun 5;19(6):e1011011. doi: 10.1371/journal.ppat.1011011 (PMC10270592; doi:10.1371/journal.ppat.1011011)
Supplement: Attachment — Submitted filename: Responses to editor and reviewers.doc [file ppat.1011011.s011.doc]

Responses to editor and Reviewers

To editor,

As with all papers reviewed by the journal, your manuscript was reviewed by members of the editorial board and by two independent reviewers. In light of the reviews (below this email), we would like to invite the resubmission of a significantly-revised version that takes into account the reviewers' comments. You should note the comments from reviewer 1 in particular. They make a number of useful suggestions in regards to providing evidence to support the conclusions drawn in this manuscript. These are suggestions and you can choose a different approach but the key here is to provide additional evidence in relation to the activity of the velvet complex in regulating IPT resistance.

**Response:** Thank you very much for giving us the opportunity to revise the manuscript, we also thank reviewers made many instructive comments to help us to improve the manuscript. As you and reviewer 1 mentioned, we added some additional evidences to make the conclusion about the velvet complex in regulating IPT resistance more solidified.

To Reviewer #1:

Focus of this manuscript entitled “The velvet family proteins mediate low resistance to isoprothiolane in Magnaporthe oryzae” where the authors performed an evolutionary adaptation experiment by exposing this fungus to antifungal drug IPT. They found three group of mutants with low and moderate group of resistance. MR-2 mutations were responsible for moderate level of resistance. However, for the low resistant mutants, interestingly they found a subunit of velvet complex MoVelB was mutated by an early stop codon. After finding this, they further studied other components of the velvet complex in Magnaporthe where they found all three components of the velvet complex, LaeA-VeA-VelB play important roles for IPT resistance. The authors conclude that the reason why the mutants of the velvet complex are resistant to IPT was down regulation of secondary metabolites or CYP450 genes. Main findings of this study are solid and sound. However, mechanism of resistance remains largely unknown, which is one of the flaws of this study.

**Response:** Yes, you are right, the detailed mechanisms are still not very clear, fortunately, we made an important step to unveil the mechanisms because the key regulator, the VelB-VeA-LaeA complex was identified.

Regarding the resistance mechanism: Authors speculate that low level IPT mechanism is due to reduced secondary metabolite levels or CYP450 genes. In Figure 7D, expression of 12 genes were tested using qRT-PCR. The authors do not even name those genes what they are and why upregulation of these 5 genes make sense?

**Response:** We are sorry, we should have added such important information. As you suggested, detailed information of these genes are shown in the S4 table. Among the 12 genes examined by RT-qPCR, only 5 of them showed consistent expression trends in Δ*VelB*, Δ*VeA* and Δ*LaeA*, and 5 genes were down-regulated in expression which is reasonable because down-regulation of these genes should weaken the ability to toxically metabolize (modify) the IPT.

Have authors overexpress VelB, VeA and LaeA to see if their overexpression results in hypersensitivity to IPT. If their assumption “reduced secondary metabolite levels” causes low IPT resistance, then overexpression of these genes or at least two of them should cause an opposite phenotype to a deletion strain. The authors only overexpress MoVelB (Fig S1D), not VeA nor LaeA. Although they mentioned that the results are not significant, what I can see from FigS1D and its quantification that IPT resistance was reduced in MoVelB overexpression. Have authors quantify overexpression of MoVelB (gene expression) in comparison to WT H08-1a. Based on what expression level we call it overexpression? Is it 3 times more, 5 times? I am just wondering if similar effect can also be observed in VeA and LaeA overexpression.

**Response:** Thank you very much for your comments. As you suggested, we overexpressed *VeA* and *LaeA* this time. Similar results were observed which were shown in Fig 6C, 6D and 6E.

Just like you said, the overexpression of *MoVelB* (gene expression) was quantified in comparison to WT H08-1a, the expression level of *MoVelB* in OE*VelB* transformants was 40-fold higher than that of wild type H08-1a (Fig S1E), and the transformants were more sensitive to IPT compared to H08-1a (Fig. S1D). When we overexpressed the *VeA* and *LaeA*, the corresponding transformants OE*VeA* and OE*LaeA* also showed increased sensitivity to IPT (Fig 6C).

It would be interesting to see overexpression of these three mutants side by side with deletions.

**Response:** Thank you very much for your suggestion. We arranged the figure as you suggested for *VeA* and *LaeA* (Fig 6C), but not for *VelB* because it was already shown in Fig S1B, S1D.

Furthermore, those genes which are drastically changed in Figure 7D should show opposite phenotype. Alternatively, the genes drastically change in opposite direction in velB knock out and overexpression strains should be the key genes of IPT resistance.

**Response:** Just as you expected, five genes showed down-regulation in Δ*VelB* knockout transformants were analyzed by RT-qPCR, all of them drastically up-regulated in OE*VelB* overexpression transformants (Fig S5A).

Line 244, “cytosolic protein LaeA……” how do the authors know that LaeA is a cytosolic protein? Do they have any evidence for this?

**Response:** We are terribly sorry for this mistake. Actually LaeA was a nuclear protein.
We revised it this time.

Figure 6E, only suggest that VeA and VelB form heterodimer via yeast two hybrid, which does not represent native forms of the protein interactions. Any CoIP would help to support their claims with these two proteins whether these interactions take place in vivo within the Magnaporthe.

**Response:** This is a very good suggestion. We made the in vivo validation of MoVelB and MoVeA interaction in *M. oryzae* by CoIP this time (Fig 6G). Meanwhile, we constructed *MoVelB* and *MoVeA* double knockout transformants Δ*VelB*Δ*VeA*, which showed low resistance to IPT and the level of resistance was not significantly different from that of Δ*VelB* or Δ*VeA* (Fig 6H).

To Reviewer #2,

In this manuscript, Meng et al., set out to determine the mechanisms of low-level resistance to the fungicide Isoprothiolane (IPT) in the rice blast fungus, Magnaporthe oryzae. The emergence of low-level resistance to IPT in blast populations is an ongoing problem in China, yet the mechanisms of low-level resistance are largely unknown, and therefore this study is novel and of significance. The manuscript is beautifully written, and the data seem solid, convincing, and mostly well-presented (however, some of the figures should be increased in size, as they were a little difficult to read in places).

**Response：**Thank you very much for your positive comments, and we made all of the figures clearer this time.

I noted only a few weaknesses in the manuscript. Firstly, precisely how the spontaneous IPT-resistant mutants were generated was not well described in the methods. As I understand it, hyphal plugs from the parental strain were inoculated on PDA followed by IPT-amended PDA. But it's not clear how many plugs were inoculated in total. Some clarification would be useful.

**Response:** Thank you very much for your suggestion, in the method section, we added the “Induction of IPT resistant mutants” to describe how the IPT-resistant mutants were generated this time .

Also, while I was comfortable with most of the conclusions in the paper, Line 249/250 states that "yeast two-hybrid assay showed that MoLaeA can directly interact with MoVeA, demonstrated that MoLae formed a heterotrimer complexes [sic]'. The yeast two-hybrid data do not, and could not, show heterotrimer formation, and the language should be changed. Ideally, these interactions would be confirmed by in vivo co-immunoprecipitation experiments, but I think this is beyond the scope of this manuscript.

**Response:** Thank you very much for your suggestion and understanding. This is also suggested by another reviewer, so we made the Co-IP for verification of interaction between MoLaeA and MoVeA. Nevertheless, We did not observe the heterotrimer structurally, we modified the heterotrimer as “complex”.

I found it difficult to read the labels on some of the figures due to their small size.

**Response:** Thank you very much for your suggestion, the images have been adjusted and were clearer this time.

Line 254: I'm not sure about the use of 'deformation' to describe the disruption of a putative LaeA/VeA/VelB complex.

**Response：**“deformation” has been modified to “disruption”

Fig.5 significance letter annotation are lowercase on G and E, and uppercase elsewhere.

**Response：**We are sorry for this mistake. We made further explanation for the significance letter annotation this time. Lowercase letters represent significance at p=0.05, uppercase letters represent p=0.01, G and E are significant at P=0.05.
